# Neurofeedback interventions for obsessive-compulsive and related disorders: Current evidence and future directions

**DOI:** 10.1016/j.jpsychires.2026.03.013

**Published:** 2026-03-11

**Authors:** Kaixi Zhang, Lucas Trambaiolli, Zhiying Zhao, Vincent Taschereau-Dumouchel, Jamie D. Feusner

**Affiliations:** a Institute of Medical Science, Temerty Faculty of Medicine, University of Toronto, Toronto, ON, Canada; b Centre for Addiction and Mental Health, Toronto, ON, Canada; c Department of Psychiatry, Temerty Faculty of Medicine, University of Toronto, Toronto, ON, Canada; d Department of Women’s and Children’s Health, Karolinska Institutet, Stockholm, Sweden; e Basic Neuroscience Division, McLean Hospital, Harvard Medical School, Belmont, MA, USA; f Centre for Cognitive and Brain Sciences, Institute of Collaborative Innovation, University of Macau, Macau SAR, China; g Department of Psychology, Université de Montréal, Montréal, QC, Canada; h Centre de Recherche de L’Institut Universitaire en Santé Mentale de Montréal, Montréal, QC, Canada

**Keywords:** Obsessive-compulsive disorder, Body dysmorphic disorder, Trichotillomania, Excoriation disorder, Hoarding disorder, Hypochondriasis, Illness anxiety disorder, Olfactory reference syndrome real-time neurofeedback, EEG-neurofeedback, fMRI- neurofeedback

## Abstract

Obsessive-compulsive and related disorders (OCRDs) are characterized by obsessions, compulsive or repetitive behaviors, hoarding and saving, or recurrent body-focused repetitive behaviors. Although pharmacological, cognitive-behavioral, or behavioral therapies provide relief for many for these conditions, a substantial proportion respond insufficiently or experience relapses. Neurofeedback (NF) enables individuals to self-modulate neural activity in real time and has been explored as a non-invasive neuromodulation strategy in obsessive-compulsive disorder (OCD), the only OCRD in which NF has been empirically tested to date. Conventional electroencephalogram-based (EEG-NF) and functional magnetic resonance imaging-based (fMRI-NF) protocols have demonstrated that patients can learn to regulate oscillatory activity or region-specific blood-oxygen-level–dependent signals, with some studies reporting symptom improvements. However, heterogeneity in targets, limited personalization, and modest clinical effects constrain conclusions regarding efficacy and scalability. This narrative review synthesizes the existing empirical evidence of NF studies to-date. In addition, it highlights the potential for decoded neurofeedback (DecNef) as a next-generation NF method. Unlike conventional NF, DecNef leverages multivoxel pattern analysis to reinforce distributed neural representations, in some cases without explicit awareness or symptom exposure, and may allow greater precision and personalization of circuit-level interventions. We discuss how DecNef can address limitations of traditional NF and outline its potential translational applications across the OCRD spectrum. By integrating current empirical findings with a forward-looking precision psychiatry framework, this review offers ideas for conceptual and methodological advances for targeting dysfunctional neural systems underlying OCRDs.

## 1. Introduction

Obsessive compulsive disorder (OCD) is a chronic and frequently disabling psychiatric disorder with a 2–3% lifetime prevalence (Rasmussen and Eisen, 1990; Ruscio et al., 2010; Sasson et al., 1997; Stein et al., 2019). OCD is characterized by the existence of obsessions, compulsions, or both (Association, 2013; Goodman, 1989). Obsessions are recurrent and persistent thoughts, images, or urges that are intrusive, unwanted, and typically cause marked anxiety or other distress. Compulsions are repetitive behaviors or mental acts that individuals feel compelled to perform in response to an obsession or in accordance with rigidly applied rules, often aimed at reducing distress or achieving a sense of “completeness” (Association, 2013; Phillips et al., 2010). OCD is associated with substantial impairments in quality of life, including occupational, social, and family functioning, as well as significant emotional distress (Lochner et al., 2014; Stein et al., 2019).

Currently, treatment options for OCD include pharmacotherapy, primarily with serotonin reuptake inhibitors (SRIs), or psychotherapy approaches, including cognitive-behavioral therapy (CBT, which typically involves exposure and response prevention (ERP) techniques) (Mao et al., 2022). Although first-line treatments can lead to symptom improvement in up to 50% of OCD patients during the acute phase (Skapinakis et al., 2016; Stein et al., 2019), relapses are common following treatment discontinuation (Gonçalves et al., 2017), and even in some cases when continuing treatment (Erzegovesi et al., 2001; Ivarsson et al., 2024). As a result, there is growing interest in developing novel interventions, including, but not limited to, novel pharmacological agents and both invasive and noninvasive neuromodulation techniques.

In OCD, dysregulation has been described in multiple neural circuits and systems, including the cortico-striato-thalamo-cortical (CSTC) circuits, fronto-limbic circuits, fronto-parietal networks, the default mode network, the salience network, sensorimotor networks, and cerebellar networks (Pauls et al., 2014). CSTC circuits have been associated with symptom severity, intrusive thoughts, and compulsive behaviors in OCD (Shephard et al., 2021). Neuroimaging studies suggest that CBT, particularly exposure and response prevention (ERP), is associated with increased activation in cognitive control regions such as the dorsolateral prefrontal cortex and parietal cortex in individuals with OCD and decreased reactivity in salience and limbic regions, consistent with the modulation of frontoparietal and salience networks involved in emotion regulation and attentional control (Becker et al., 2024; Machado-Sousa et al., 2025). However, the circuit-level specificity remains limited and variable across individuals (Bennett et al., 2023; Moody et al., 2017; Nakao et al., 2005). While some task-based fMRI studies in OCD have reported altered brain activation following ERP interventions, such as in circuits implicated in inhibitory control (Stephenson et al., 2024), the extent to which these neuroplastic changes reliably mediate symptom reduction remains unclear. Variability in task paradigms, imaging modalities, and individual differences may partly account for inconsistent findings across neuroimaging studies (Becker et al., 2024; Figee et al., 2011; Moody et al., 2017; Stephenson et al., 2024).

Noninvasive neuromodulation methods, such as repetitive transcranial magnetic stimulation (TMS) and transcranial direct current stimulation (tDCS), induce electric currents and modulate cortical excitability, respectively (Moshfeghinia et al., 2025; Steuber and McGuire, 2023). These have primarily been studied in OCD (Fitzsimmons et al., 2022; Rapinesi et al., 2019). Repetitive TMS has demonstrated clinical efficacy with significant symptom reduction compared to sham controls (Carmi et al., 2019), leading to the FDA clearance of deep TMS systems for OCD treatment, and the CE Mark approval in Europe enables clinical use across multiple member countries (Cotovio, Ventura, Rodrigues da Silva, Pereira and Oliveira-Maia, 2023). Response rates are estimated at approximately 38–55% with protocols typically targeting the dorsomedial prefrontal cortex or ACC using high-frequency stimulation (Roth et al., 2021). Additional targets such as the supplementary motor area (SMA) and dorsolateral prefrontal cortex (DLPFC) (Dehghani-Arani et al., 2024; Rehn et al., 2018; Zhou et al., 2017) have also shown promising results in reducing OCD symptoms, likely through modulation of fronto-striatal circuits (Mantovani et al., 2010). However, their spatial precision and individual tailoring for OCD patients at this point are limited. Invasive neuromodulation like deep brain stimulation targets specific neural sites (most within, or connected to, CSTC circuits) to alleviate OCD symptoms. It is typically reserved for severe, treatment-refractory cases due to potential surgical and hardware-related complications, stimulation-induced side effects, and other adverse outcomes (Mar-Barrutia et al., 2021).

Neurofeedback (NF) is also a neuromodulation technique, as its goal is to alter neuronal activity directly, although this process is achieved endogenously rather than exogenously through electrical or magnetic stimulation (L. R. Trambaiolli et al., 2021). NF enables individuals to modulate specific neural patterns in real-time feedback. This method can directly target dysregulated circuits and can be personalized (Stoeckel et al., 2014). This possibility for precision, combined with its noninvasiveness, positions NF as a promising next-generation intervention to address circuit- or systems-level dysfunctions in psychiatric disorders in a more individualized and dynamic manner (Pindi et al., 2022).

NF is a non-invasive, closed-loop brain training technique that most likely involves learning mechanisms (Sitaram et al., 2017). As a form of biofeedback, it relies on the real-time measurement and representation of neural activity, typically through visual, auditory, or other sensory modalities, to enable individuals to self-regulate neural processes presumed to underlie specific behaviors or neuropathological conditions (Shibata et al., 2019).

**Fig. 1.**
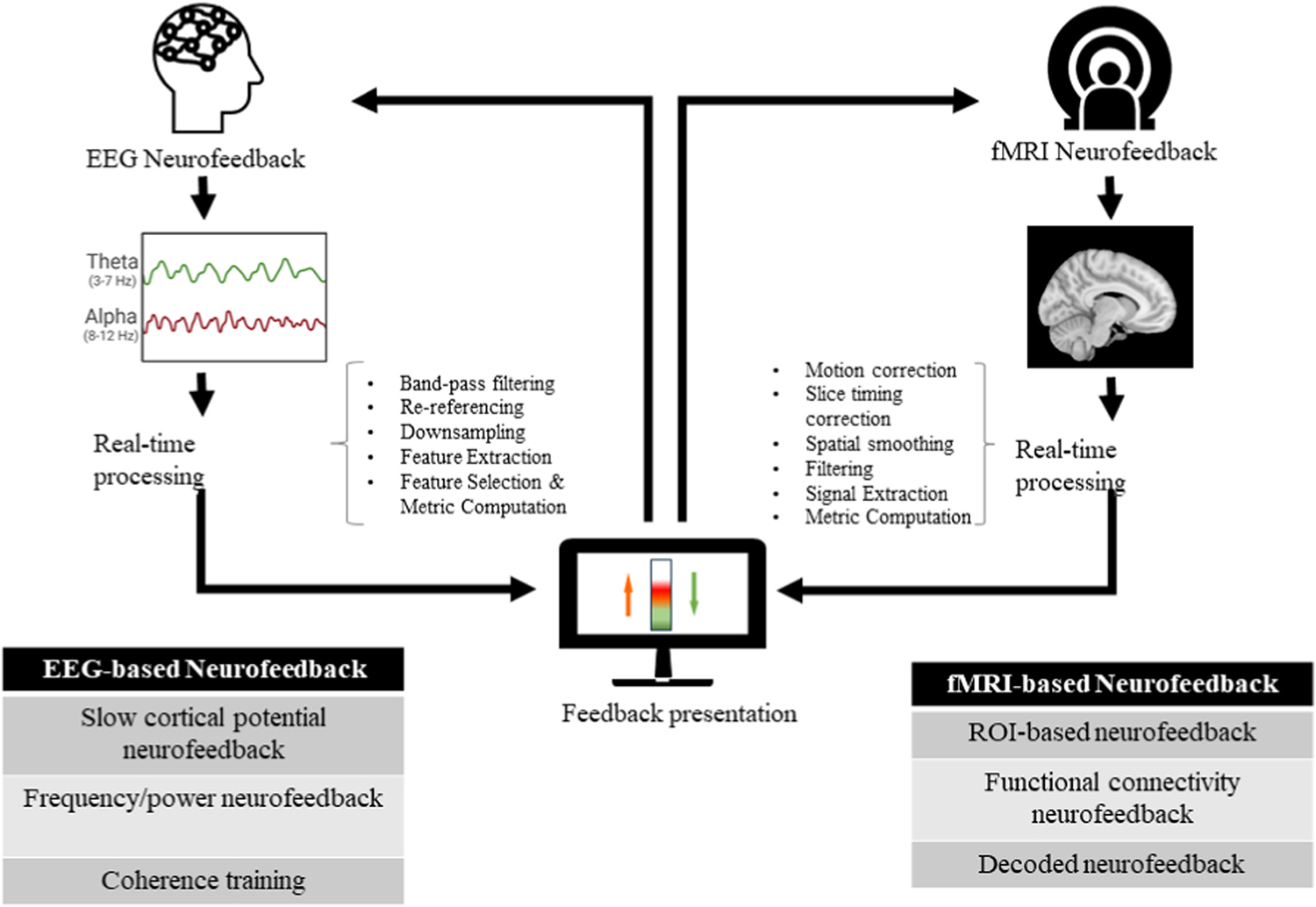
Overview of EEG-based and fMRI-based NF procedures and processing workflow. (a) Participants undergo either EEG recording (left) or fMRI scanning (right) to capture real-time brain signals. Real-time processing is performed on the acquired data. For EEG-NF, this typically involves steps such as band-pass filtering, re-referencing, downsampling, feature extraction and metric computation. For fMRI-NF, preprocessing typically includes motion correction, slice timing correction, spatial smoothing, signal filtering, and extraction of region-specific BOLD signals. (Specific preprocessing pipelines may vary across studies.) Processed signals are translated into feedback displays (e.g., visual indicators such as arrows or thermometers), which are presented to participants in real time to guide self-regulation of brain activity. Three common EEG-based NF protocols include slow cortical potential NF, frequency/power NF and coherence NF (Marzbani et al., 2016; Omejc et al., 2019). The common fMRI-based NF protocols, which vary by the feedback signal used, are signal amplitude-based, functional connectivity-based, and decoded NF. Additionally, hybrid approaches such as EEG-fMRI fingerprint decoding may be used to train a machine learning classifier of brain activity pattern, sometimes called as ‘decoder,’ that maps EEG features to corresponding fMRI activity. Processed signals are translated into feedback displays (e.g., visual indicators such as arrows or thermometers), which are presented to participants in real time to guide self-regulation of brain activity.

Conventional NF typically relies on training individuals to modulate activity within predefined brain regions or frequency bands, most often using electroencephalogram (EEG) or fMRI signals such as BOLD signal change in specific regions or functional connectivity (Taschereau-Dumouchel et al., 2022) (Fig. 1). Participants receive real-time feedback about their ongoing signal amplitude-based or functional connectivity-based brain activity and are encouraged to voluntarily regulate it to achieve a desired mental or neural state (Taschereau-Dumouchel et al., 2022). This approach has been used to target broad neural patterns associated with symptom relief across various psychiatric conditions (Heunis et al., 2020; Pindi et al., 2022). Notably, even when NF targets specific regions, its effects often extend to broader neural circuits, likely through modulation of anatomically interconnected structures (L. Trambaiolli et al., 2024). More recently, decoded neurofeedback (DecNef), a multivariate representational approach, has been proposed as a potential next-generation extension of the conventional NF (Taschereau-Dumouchel et al., 2022).

As neuroimaging studies have identified characteristic neural activity patterns, such as hyperactivity in CSTC circuits, that correlate with specific clinical features of OCD (with a sparser but growing knowledge base in other OCRDs), this concept has provided a rationale for exploring NF as a therapeutic strategy targeting dysfunctional circuits. For these reasons, research has explored neuroimaging-based NF as a therapeutic strategy for OCD (Shephard et al., 2021; Zafarmand et al., 2022).

The goal of this narrative review is to assess the existing evidence for the efficacy of EEG- and fMRI-based NF studies and synthesize the current state of NF interventions for treating OCD. Further, it discusses whether multivariate approaches, such as DecNef, may address the identified mechanistic limitations. Discussion of DecNef and of OCRDs is therefore explicitly conceptual and prospective, with the goal of outlining mechanistic considerations and future research directions rather than evaluating established clinical efficacy. Throughout, we emphasize the importance of causal target identification and mechanistic specificity in advancing neurofeedback as a viable intervention for OCD and related conditions. While prior systematic reviews and meta-analyses have evaluated the clinical efficacy of neurofeedback in OCD (e.g., Ferreira et al. (2019); Zafarmand et al. (2022)), these have primarily focused on quantitative symptom outcomes and study quality. The present review differs in several important respects. First, we extend the discussion beyond OCD to consider potential translational targets across the broader OCRD spectrum. Second, we introduce and conceptually evaluate DecNef as a next-generation approach that may overcome limitations of conventional protocols. By situating neurofeedback within a precision psychiatry perspective, this review aims to provide a forward-looking synthesis rather than solely a summary of existing trials.

## 2. Method

This narrative review was conducted in accordance with the SANRA (Scale for the Assessment of Narrative Review Articles) guidelines (Baethge et al., 2019). To ensure a structured approach, the PICO framework was employed: (i) Population (P): individuals diagnosed with OCD based on DSM-5 or ICD criteria; (ii) Intervention (I): the focus was on both EEG-NF and fMRI-NF techniques; (iii) Comparison (C): where applicable, interventions were compared against sham procedures, standard care, or alternative treatments; (iv) Outcome (O): primary outcomes of interest included symptom improvement, remission rates, cognitive performance, global functioning, and quality of life.

A comprehensive literature search was conducted across major databases including Embase, PubMed/MEDLINE, Google Scholar, and APA PsycINFO, to May 1, 2025. Search terms combined “OCD” OR “obsessive compulsive disorder” OR “obsessive-compulsive disorder” combined by AND with “neurofeedback” OR “neuro feedback” OR “EEG biofeedback” OR “fMRI biofeedback” OR “neurotherapy” OR “EEG neurofeedback” OR “EEG neuro feedback” OR “fMRI neurofeedback” OR “fMRI neuro feedback”. Only English-language studies published in peer-reviewed journals were considered. In addition to database searches, the reference lists of relevant studies and reviews were also screened to identify additional eligible citations. Original empirical studies and case reports examining NF interventions in OCD were eligible for inclusion. Reviews and meta-analyses were consulted for contextual interpretation and citation tracking, but were not included as primary data sources. Following screening and eligibility assessment, nine original research studies met the inclusion criteria and were included in the narrative synthesis. Extracted data included study design, sample characteristics, neurofeedback modality, neural targets, outcome measures, and follow-up duration. Findings are summarized narratively and presented in Table 1.

## 3. Results

### 3.1. Overview of included studies

A total of nine empirical studies met the inclusion criteria, including EEG-based and fMRI-based NF studies (see Table 2). All studies examined individuals with OCD or elevated obsessive-compulsive symptoms. Sample sizes were generally small, ranging from single-case reports to small clinical cohorts, and study designs varied substantially across investigations. Control conditions were heterogeneous and included waiting-list controls, sham or minimal-feedback conditions, or uncontrolled pre–post designs.

Across studies, NF protocols targeted neural activity or connectivity patterns implicated in OCD-related circuits, with EEG-based approaches focusing on oscillatory features and qEEG-guided abnormalities, and fMRI-based protocols primarily targeting orbitofrontal and related fronto-striatal regions. Clinical outcomes were most commonly assessed using standardized measures such as the Yale–Brown Obsessive Compulsive Scale (Y-BOCS), although outcome definitions and reporting practices varied.

Several studies reported reductions in OCD symptom severity following NF training; however, heterogeneity in methodology, limited sample sizes, and inconsistent follow-up assessments preclude quantitative synthesis. Accordingly, findings are presented descriptively in the following sections, organized by NF modality.

### 3.2. Electroencephalogram (EEG)-Based neurofeedback

EEG-based neurofeedback (EEG-NF) has been explored as an intervention for OCD, with advantages including accessibility, noninvasiveness, and high temporal resolution. EEG-NF approaches in OCD can be broadly categorized into (1) standardized frequency-band protocols (e.g., alpha-theta or sensorimotor rhythm training), which target predefined oscillatory bands, and (2) individualized protocols guided by quantitative EEG (qEEG) assessments, in which training targets are selected based on patient-specific electrophysiological abnormalities. Across included studies, both standardized and qEEG-guided approaches were represented, with modulation of alpha, theta, beta, or sensorimotor rhythms using scalp-EEG recordings. Clinical outcomes were commonly assessed using standardized symptom measures such as the Y-BOCS.

Several studies reported reductions in obsessive and compulsive symptoms following EEG-based NF. In a small randomized study, qEEG-guided NF was associated with greater symptom reduction than a waiting-list control condition (Barzegary et al., 2011), while additional pre– and post- and case-series designs described symptom reductions after training (Hammond, 2003; Sürmeli and Ertem, 2011). However, sample sizes were small, and the magnitude and consistency of clinical effects varied across studies.

Neural outcomes were reported less consistently. Some studies observed changes in targeted oscillatory features or coherence patterns following training, including trends toward normalization of baseline abnormalities (Hawley et al., 2021; Kopřivová et al., 2013). In other cases, clinical symptom improvement occurred without statistically significant changes in EEG metrics (Barzegary et al., 2011), and associations between neural modulation and symptom change were not consistently demonstrated.

Methodological limitations were common. Protocols varied substantially in target selection, session structure, and feedback parameters, limiting comparability across studies (Hammond, 2003; Kopřivová et al., 2013). Control conditions ranged from sham or minimal-feedback paradigms to uncontrolled pre–post designs, and blinding procedures were often absent. In addition, follow-up assessments were inconsistently reported, precluding firm conclusions about the durability of the effects.

Taken together, current EEG-NF evidence in OCD suggests preliminary feasibility and possible symptom benefit, but small samples, heterogeneity in design, and limited mechanistic clarity restrict conclusions regarding efficacy and neural specificity.

### 3.3. Functional magnetic resonance imaging-based neurofeedback

FMRI-NF studies in OCD have targeted regions within CSTC circuits, including the OFC, and anterior prefrontal cortex (aPFC; BA10), as well as the anterior insula, a region implicated in salience and affective processing (Buyukturkoglu et al., 2015; Gonçalves et al., 2017; Rance et al., 2023; Dustin Scheinost et al., 2014). Early work in subclinical samples with high levels of contamination-related anxiety demonstrated that participants could learn to modulate OFC activity, accompanied by changes in functional connectivity and reductions in anxiety-related measures (Hampson et al., 2012). Using individually localized OFC as the regulation training target, results suggested alterations in widespread global connectivity (including OFC) and reduced contamination anxiety (D Scheinost et al., 2013). These findings were later discussed in the context of OCD neurofeedback applications by Gonçalves et al. (2017).

In a small-sample pilot study involving individuals with OCD, participants demonstrated the ability to modulate OFC activity during neurofeedback training, with associated reductions in Y-BOCS scores (Dustin Scheinost et al., 2014). A later double-blind randomized study using a yoked-feedback control condition found modest but statistically significant reductions in Y-BOCS scores over time in the active neurofeedback group compared to controls, although improvements in OFC regulation did not differ significantly between groups (Rance et al., 2023).

In addition to OFC-focused protocols, anterior insula modulation has been explored in a small pilot study. Participants demonstrated variable ability to down-regulate insula activity, with preliminary improvements in distress and emotion regulation (Buyukturkoglu et al., 2015).

Across studies, sample sizes were small in clinical OCD cohorts, and control conditions were limited (Dustin Scheinost et al., 2014) or absent (Buyukturkoglu et al., 2015) in several investigations. Outcome measures varied, and follow-up assessments were inconsistently reported. While neural modulation within targeted regions was generally demonstrated, the casual relationships between neural regulation and clinical improvement remain incompletely characterized. Overall, current fMRI-NF evidence in OCD suggests feasibility and possible symptom benefit, but methodological constraints and limited replication preclude firm conclusions regarding efficacy and mechanistic specificity.

### 3.4. Meta-analyses of fMRI-based NF studies in OCD studies

Two meta-analyses have synthesized evidence regarding the efficacy of NF for OCD. Zafarmand et al. (2022) reported a significant advantage of NF over control conditions in 9 studies, with a large effect size on Y-BOCS scores (MD = −6.82, 95% CI [−9.03, −4.60]), although substantial heterogeneity (I^2^ = 92.7%) and methodological limitations were noted. Similarly, Ferreira et al. (2019) found medium-to-large within-group effect sizes (ES = 2.47) favoring NF interventions but highlighted critical design flaws, including small samples, high risk of bias, and inconsistent control conditions (e.g., only 1/5 trials used sham-NF). Both meta-analyses concluded that further high-quality RCTs are essential to establish efficacy and optimize protocols.

### 3.5. Summary of existing studies in OCD

Despite the relatively small body of evidence, studies described in this section have shown promise in reducing OCD symptom severity, including contamination-related anxiety and the effects appear to normalize activities in the underlying neural circuits. Compared to EEG-NF training protocols, fMRI studies also demonstrated greater flexibility in identifying and targeting individualized brain activity patterns by combining anatomical reference with individualized functional localizers to define the training regions. The efficacy of fMRI-NF in treating other OCD and OCRD symptoms remains to be evaluated.

## 4. Limitations and conceptual challenges

Although preliminary findings suggest that both EEG- and fMRI-based neurofeedback (NF) is feasible in OCD and may be associated with symptom improvement in some individuals, the current evidence base is constrained by several methodological limitations that restrict interpretability and generalizability.

Firstly, most studies have involved small samples (Ferreira et al., 2019; Zafarmand et al., 2022), ranging from single cases to modest cohorts. Such designs reduce statistical power, inflate effect size estimates, and limit generalizability. Secondly, control conditions have been heterogeneous or frequently absent (Ferreira et al., 2019); many studies rely on pre–post comparisons or waiting-list controls rather than active sham paradigms. Even when sham conditions are used, implementation varies, complicating cross-study comparisons. Blinding procedures are inconsistently reported. Fourthly, follow-up assessments are often limited or absent, limiting conclusions regarding durability and the generalizability of effects in the real world. Furthermore, protocols differ substantially in target selection, training duration, feedback modality, and outcome measures. This heterogeneity prevents clear identification of the components most critical for clinical benefit.

Beyond methodological concerns, a central conceptual challenge relates to target validity and causal relevance. Conventional NF protocols typically select neural targets based on abnormalities identified in correlational or case-control neuroimaging or electrophysiological studies. However, neural patterns that differ between individuals with OCD and controls or that correlate with symptom severity do not necessarily represent causal drivers of symptom maintenance. Such signals may reflect downstream consequences, compensatory processes, state-dependent effects (e.g., heightened arousal or attentional engagement), or even epiphenomena rather than mechanisms that, when modified, produce sustained symptom change.

Consistent with this concern, the relationship between neural modulation and clinical improvement remains unclear across many studies. In some EEG-based investigations, symptom reduction has occurred without statistically robust normalization of the targeted oscillatory feature (Barzegary et al., 2011; Kopřivová et al., 2013; Rance et al., 2023). In fMRI-based protocols, participants may successfully modulate regional activity (e.g., orbitofrontal or insular signals), yet the extent to which such modulation alters the cognitive or affective computations underlying obsessions and compulsions is not clearly demonstrated. This neural–clinical dissociation raises the possibility that observed improvements may partly reflect nonspecific factors such as structured engagement, expectancy effects, or broad regulatory strategies rather than targeted modification of causal circuitry. Such off-target effects may nevertheless prove beneficial, yet establishing the mechanisms underlying them would be important for optimizing their clinical impact.

A related issue concerns the level of neural representation at which conventional NF operates. Many NF protocols tested to date, in general, reinforce coarse proxies, such as mean regional activation, spectral power within a frequency band, or connectivity strength between regions. While informative, these measures may only indirectly index the psychological constructs most relevant to OCD, including threat valuation, uncertainty intolerance, maladaptive habit formation, or dysfunctional error monitoring. Moreover, given the heterogeneity of OCD symptom dimensions, a single regional or frequency-based target may not correspond to the same underlying process across individuals. Further, multiple systems may need to be modulated for more complete symptom reduction.

Taking together, these limitations suggest that advancing NF for OCD will require improved methodological rigor and more precise frameworks for target selection. In particular, approaches that engage symptom-relevant neural representations more directly and clarify the mechanistic link between neural modulation and behavioral change may offer a promising direction for future development.

## 5. Conceptual extensions and future directions

### 5.1. Potential targets for OCD

Future research in conventional NF for OCD should prioritize refining target selection and improving experimental design.

Rather than reinforcing broadly defined regional abnormalities, protocols may benefit from aligning training targets more closely with specific symptom dimensions, such as threat overestimation, uncertainty intolerance, or maladaptive habit formation. Integrating task-based paradigms that isolate these processes during target identification may improve specificity.

In addition, future trials should incorporate adequately powered randomized controlled designs with active sham conditions and longitudinal follow-up. Mechanistic analyses assessing whether changes in targeted neural signals mediate symptom improvement will be critical for clarifying causal relevance. Since these may not always be evident, exploratory analyses can play a role in uncovering unexpected yet potentially informative effects. Multimodal tracking approaches, including behavioral tasks and ecological momentary assessment, may further help link neural modulation to real-world symptom change.

### 5.2. Potential targets for OCD related disorders

To date, NF has only been tested in OCD. Emerging neuroimaging findings in obsessive-compulsive related disorders (OCRDs) suggest potential avenues for hypothesis-driven extension.

In body dysmorphic disorder (BDD), altered activity has been observed in visual processing regions implicated in distorted perceptual representations (Feusner et al., 2011; Wang et al., 2010; Wong et al., 2022), as well as in fronto-striatal and default mode network circuits associated with compulsive behaviors and maladaptive self-referential evaluation (Fang et al., 2022).

In hoarding disorder, abnormal activation has been observed in both visual and decision-making networks (Levy et al., 2021; Woody, Kellman-McFarlane and Welsted, 2014; Zakrzewski et al., 2020). Trichotillomania and excoriation disorder have been associated with dysregulation in motor control and striatal circuits(Huggins et al., 2020; Odlaug et al., 2016; Wabnegger and Schienle, 2019; Chamberlain et al., 2005).

While these findings suggest candidate neural systems that could, in principle, be targeted using neurofeedback, such applications remain speculative. No neurofeedback trials have yet been conducted in these populations. Direct translation from correlational neuroimaging findings to intervention targets should be approached cautiously.

Accordingly, extension of neurofeedback to OCRDs should proceed incrementally, beginning, e.g., by targeting neural circuits whose disruption has been robustly demonstrated across studies, or, alternatively, by targeting neural circuits to enhance neural function and achieve compensatory effects with mechanistic plausibility. Rigorous, controlled testing of NF procedures, along with mechanistic validation, is warranted before they are ready for broader clinical testing.

## 6. Ethical considerations of NF

Ethical issues in conventional neurofeedback primarily concern informed consent, participant autonomy, and appropriate clinical use. These considerations are particularly important when working with vulnerable populations, such as children or individuals with severe psychiatric symptoms (Chaudhary, 2025). Participants and guardians should be clearly informed about the experimental status of many NF applications in OCD, potential benefits, and possible risks. Concerns also extend to non-therapeutic enhancement uses, which raise broader questions regarding fairness, privacy, and the nature of personal achievement. Although conventional EEG- and fMRI-based NF are generally considered low-risk and non-invasive, mild transient side effects such as fatigue, headache, emotional discomfort, or temporary symptom exacerbation have been reported (Rahmani et al., 2023). Systematic monitoring and transparent reporting of adverse events remain essential to ensure ethical and responsible implementation.

## 7. A potential next-step for NF for OCD: DecNef, and its practical considerations

The limitations outlined above highlight the need for NF approaches that engage symptom-relevant neural processes, with greater capacity to capture the multivariate complexity of distributed neural responses and clearer mechanistic grounding. DecNef represents one such next-generation framework. Rather than reinforcing coarse regional signals or frequency bands, DecNef operates at the level of distributed multivoxel patterns associated with specific cognitive or affective representations (Shibata et al., 2019).

**Fig. 2.**
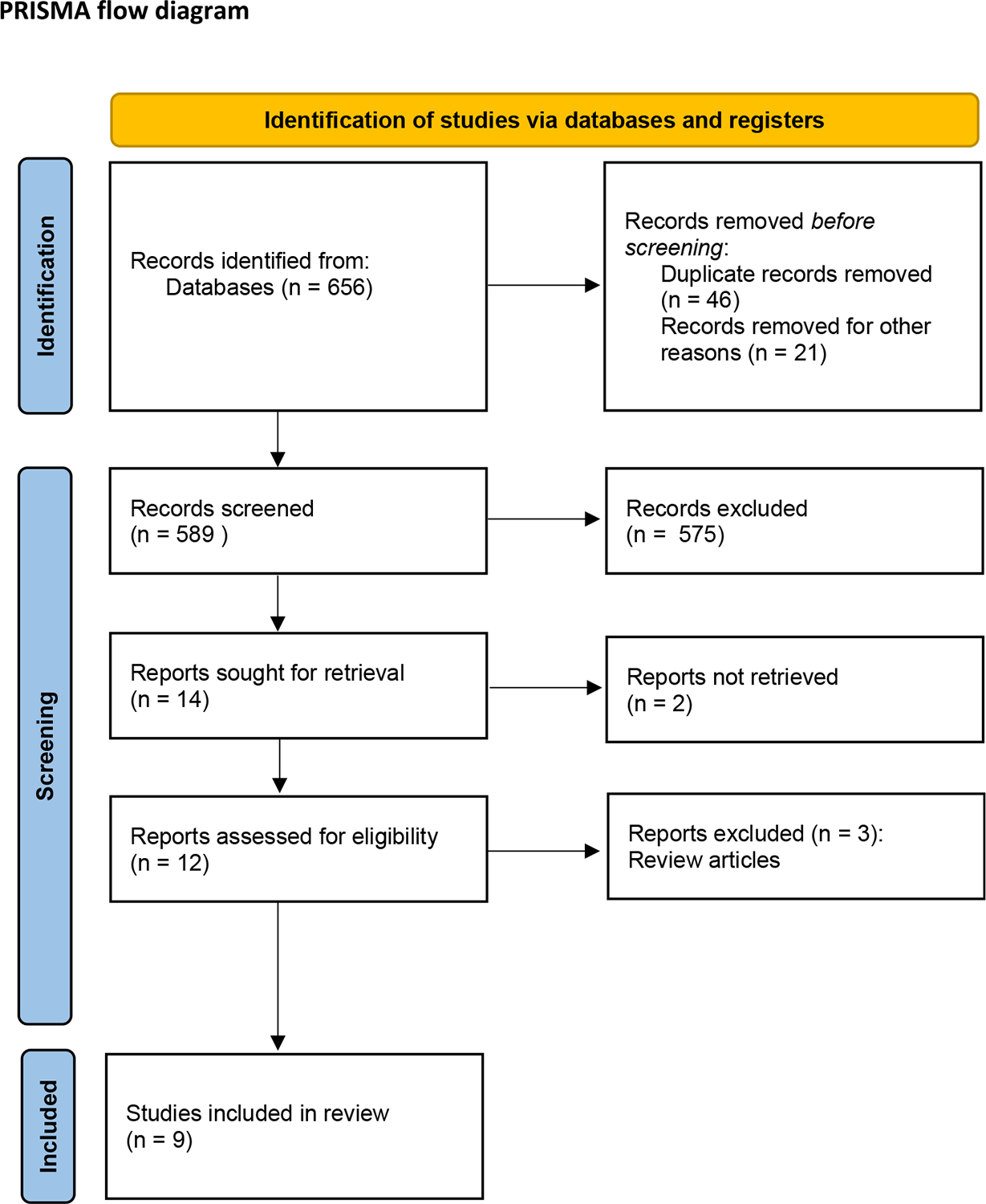
PRISMA-based flow diagram of study selection for the narrative review.

**Fig. 3.**
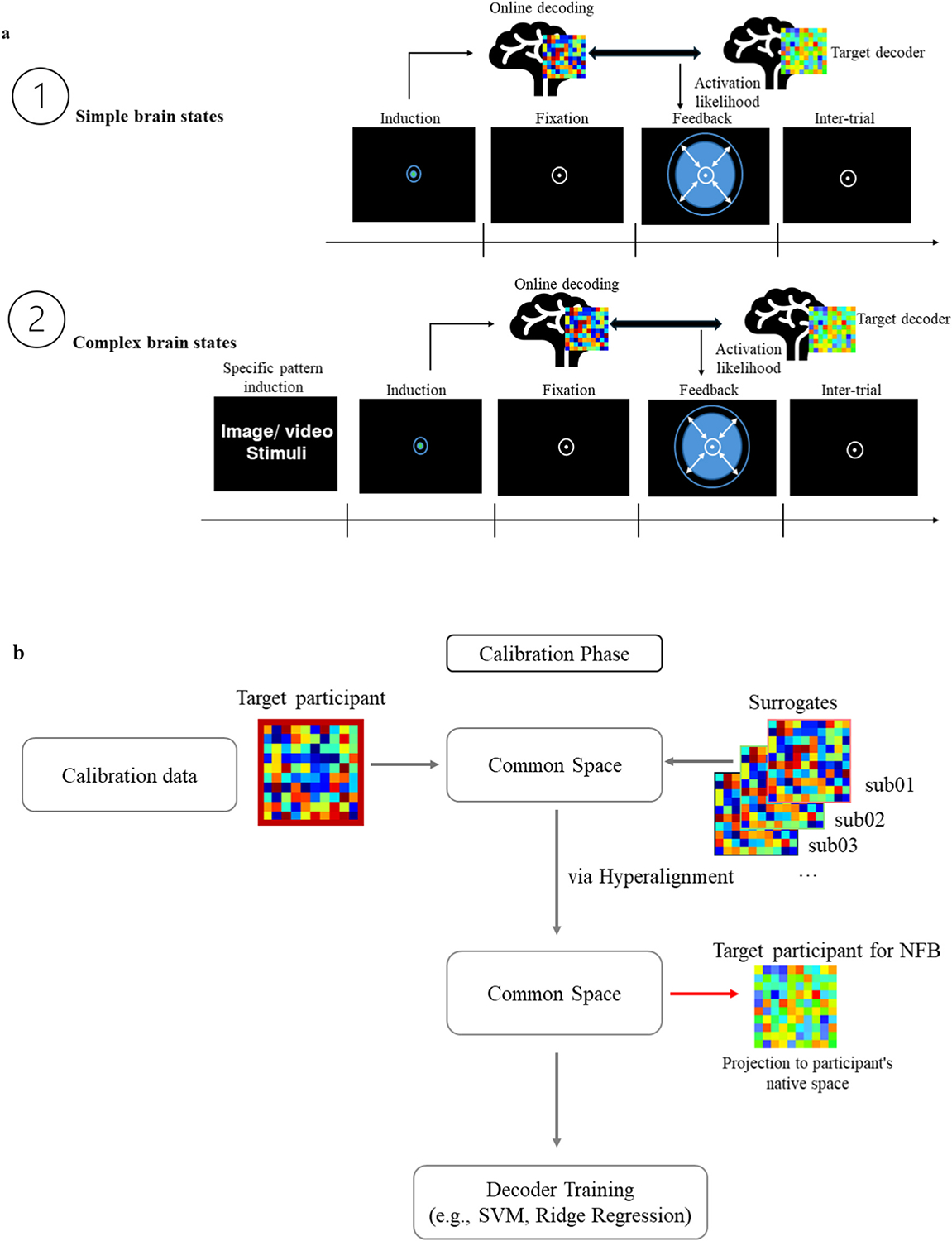
Workflow of Decoded Neurofeedback (DecNef) incorporating decoder construction and surrogate alignment in the participant’s native space. a) Two representative DecNef trial structures are shown. Both have some similar design features: during the “Induction” period, participants are instructed to modulate or change their brain activity in order to maximize the feedback received later in the trials. Activation likelihood refers to the probability that the participant’s current brain activity during the induction period represents the target category, as estimated by the decoder using, e.g., logistic regression. The likelihood is then fed back to participants in the form of a disc; the larger the disc is, the larger the obtained reward. (1) In studies targeting “simpler” brain states such as early visual areas, for example (Amano et al., 2016; Cortese et al., 2017) the induction period typically uses simple geometric stimuli, such as colored disks or colored vertical grating patterns, to facilitate pattern-specific activation without complex perceptual processing. (2) For applying DecNef on more complex brain states (and in situations for example, to minimize participant exposure to aversive stimuli), brief image or video stimuli, for example, could be presented prior to the induction period (Shibata et al., 2016). This approach aims to prime activation in target brain patterns during induction. b) Data projection from surrogates to the target participant’s native space. Functional hyperalignment is first applied to a set of surrogate participants who undergo the same task (e.g., images viewing, movie viewing), during the calibration phase. Their voxel-wise activation patterns are mapped into a shared common representational space. After performing functional hyperalignment, the functional data can be projected back from the common space into the native voxel space of the target participant, and the decoder is then trained within that native space. This enables individualized real-time NF without necessarily requiring the target participant to undergo the stimuli viewing procedure themselves. (Taschereau-Dumouchel et al., 2022).

DecNef includes two core steps: offline decoder construction and an online induction-feedback loop. In the first step, machine learning algorithms are trained, typically on fMRI data collected during specific tasks, to identify multivoxel patterns that reliably represent a cognitive or emotional state. In the second step, the induction phase, participants receive real-time feedback based on the activation likelihood of the machine learning algorithm trained in the decoder construction phase. It indicates the likelihood that the current brain activity corresponds to the target pattern. This information is thought to facilitate a form of reinforcement learning through implicit reward mechanisms (Fig. 2) (Shibata et al., 2019) (see Fig. 3).

Decoder construction in DecNef can be implemented using either whole-brain (Berman et al., 2024; Debettencourt et al., 2015) or region-of-interest (ROI)-based approaches. Evidence from perceptual learning (Cortese et al., 2017; Li et al., 2024; Margolles et al., 2024; Shibata et al., 2011; Taschereau-Dumouchel et al., 2021; Wang et al., 2021) and fear-conditioning (Koizumi et al., 2016; Taschereau-Dumouchel et al., 2018) paradigms demonstrates that DecNef can modify neural representations and associated physiological responses without explicit exposure to aversive stimuli. DecNef has been shown to reduce conditioned fear responses and amygdala reactivity in subclinical populations (Koizumi et al., 2016; Taschereau-Dumouchel et al., 2018), including in double-blind randomized designs (Cushing et al., 2024; Koizumi et al., 2016; Taschereau-Dumouchel et al., 2018). These findings suggest that representationally targeted neurofeedback can alter affective processing even in the absence of conscious strategy use or direct symptom confrontation.

Conceptually, DecNef addresses several limitations identified in conventional neurofeedback. By targeting distributed representations rather than mean regional activation (Shibata et al., 2011; Taschereau-Dumouchel et al., 2021), it may more directly engage the neural patterns underlying specific cognitive or affective processes. The reliance on implicit learning may reduce nonspecific cognitive strategy effects and minimize distress associated with symptom-provocation paradigms. In this way, it could hypothetically have a role for individuals with OCD or OCRDs who cannot tolerate exposure-based treatments. In addition, the use of individualized or functionally aligned decoders offers a potential route toward greater personalization while maintaining mechanistic specificity.

However, DecNef has not yet been empirically tested in OCD. Its proposed advantages remain theoretical within this context and require rigorous experimental evaluation. Determining whether multivariate neurofeedback yields superior clinical or mechanistic outcomes compared to conventional approaches will require carefully controlled trials incorporating mediation analyses and long-term follow-up.

## 8. Conclusions

Neurofeedback has emerged as a promising, although still preliminary, interventional approach for treating OCD. Existing EEG- and fMRI-based neurofeedback studies in OCD suggest that neural activity modulation is feasible and may be associated with symptom improvement in some individual (Barzegary et al., 2011; Ferreira et al., 2019; Rance et al., 2023). However, the current evidence base remains limited by small sample sizes, heterogeneous protocols, variable control conditions, and inconsistent relationships between neural modulation and clinical outcomes. As such, the clinical efficacy and durability of conventional neurofeedback interventions in OCD have yet to be firmly established.

A central challenge highlighted by the present review concerns target selection and causal relevance. Many neural abnormalities identified in OCD derived from correlational neuroimaging studies and may reflect downstream or state-dependent processes rather than primary mechanisms sustaining symptoms (Pauls et al., 2014; Stein et al., 2019). Neurofeedback interventions that reinforce such correlates may therefore yield limited, transient, or no effects. Advancing neurofeedback as a mechanistically grounded treatment will require frameworks that directly engage symptom-relevant neural representations and incorporate designs that support causal inference.

DecNef offers a conceptually distinct, future-oriented framework that may help address some of these challenges by operating at the level of distributed neural representations and supporting implicit learning. Although DecNef has not yet been empirically applied to OCD, evidence from related domains demonstrates the feasibility of representationally targeted neurofeedback and provides a rationale for its investigation in this context (Shibata et al., 2011; Taschereau-Dumouchel et al., 2018). Importantly, the potential advantages of DecNef remain hypothetical at this point and require rigorous empirical testing.

In conclusion, the current state of neurofeedback research in OCD reflects both promise and substantial uncertainty. Future progress will depend on integrating neurofeedback with mechanistic models of symptom generation, prioritizing causal target validation, and implementing well-controlled experimental and clinical studies. Through such efforts, NF, whether conventional or decoded, may ultimately contribute to more precise and effective interventions for OCD.

## Figures and Tables

**Table 1:** SANRA (Scale for the Assessment of Narrative Review Articles) quality assessment of the present narrative review.

SANRA Item	How Addressed	Location	Score
Justification of importance	Highlighted treatment resistance in OCRDs and lack of circuit-specific NF reviews	Introduction	
Aims	Clearly defined aims to synthesize EEG-NF, fMRI-NF, and DecNef	End of Introduction	
Literature search	Described database search and keywords	Methods	
Referencing	Included recent reviews and empirical trials	Methods	
Scientific reasoning	Critically evaluated sample sizes, heterogeneity, sham controls	Discussion	
Data presentation	Included summary tables and conceptual model figure	Table, Figure	

**Table 2:** Summary of published EEG- and fMRI-based neurofeedback (NF) studies targeting obsessive-compulsive disorder (OCD).

First Author (Year)	Study Type	Modality	Sample Size	Target Region/Frequency	Key Findings Summary	Protocol Specificity (Personalized and/or Subtype-Specific)
**EEG-NF studies**						
Hammond (2003)	Case-series	qEEG-guided NF	2 OCD patients	Case 1: Depressive phase: Fp1/F3 (inhibit θ/α, enhance 12–18 Hz) OCD phase: T5–P3 (inhibit 6.5–11 Hz α, enhance 15–18 Hz) Case 2: Fz–Cz/Cz–C4 (inhibit 19–25 Hz β, enhance 12–15 Hz) F7–F8 (inhibit 7–11 Hz α, enhance 13–16 Hz)	Significant therapeutic effects: Y-BOCS improvement: 84.6% (Case 1)/72% (Case 2) Padua Inventory improvement (Sanavio, 1988): 88.9% (Case 1)/88.7% (Case 2) Sustained benefits: stable effects at 15- and 13-month follow-up	Personalized
Sürmeli and Ertem (2011)	Case-series	α-θ NF	36 medication-resistant OCD patients	Frontal (F3/Fz/F4), Centro-parietal-temporal (C4-P4/P4) Inhibit θ/α/β excess; Reward SMR; Inhibit α coherence	Preliminary success extending anxiety/PTSD protocol to OCD Based on standardized scales: Y-BOCS total score ↓21.53 points (severe to mild symptoms; p <0.01) Clinical Global Impression-Severity scale (Guy, 1976): ↓4.19 points (“severely ill”→”borderline ill”; p <0.01) Minnesota Multiphasic Personality Inventory Psychasthenia scale (Graham, 1978): ↓15.41 points (n =17; p <0.01) Clinical outcomes: 91.7% (33/36) showed symptom improvement 26-month follow-up: 52.8% (19/36) maintained gains, 13.9% (5/36) relapsed	Non-personalized
Barzegary et al. (2011)	RCT	qEEG-guided NF	12 OCD patients (4 NF training/4 SRI/4 waitlist control)	Personalized qEEG abnormalities	NF group showed significant reduction in compulsions vs. waiting list (p < 0.05), comparable to SRI based on Padua Inventory: Significant reduction in obsessive symptoms vs. waitlist (*p = 0.021*) Significant reduction in compulsive symptoms vs. waitlist (*p = 0.057*) No significant difference vs. SRI drug therapy (obsessive: *p = 0.886*; compulsive: *p = 0.2*)	Personalized (qEEG subtypes)
Kopřivová et al. (2013)	RCT	ICA-based NF	18 OCD patients (8 NF/10 sham)	Anterior cingulate, insula, frontal gyri Individualized (3–8 Hz or 13–16 Hz)	NF group had greater compulsion reduction (p = 0.015); high δ/β predicted poor outcome Based on Y-BOCS: NF significantly reduced compulsions (% improvement: 56% vs 21%, *p = 0.015*) No group difference in obsessions (*p = 0.863*) or total Y-BOCS (*p = 0.089*) EEG predictors: Pretreatment ↑ δ (1–6 Hz) in ACC predicted worse outcome Pretreatment ↑ β (18.5–21 Hz) in OFC correlated with posttreatment severity	Personalized (ICA source)
Deng et al. (2014)	RCT	Multiband NF	72 OCD patients (37 NF + CBT + SRI/35 CBT + SRI)	α, SMR, θ bands	Based on Y-BOCS & RBANS (J. Wang et al., 2009), NF + CBT + SRI group had 86.5% response rate (vs 62.9% control, *p = 0.021*) with ≥50% Y-BOCS reduction Faster symptom relief: Significant Y-BOCS improvement by Week 6 (*p = 0.008*) Cognitive enhancement: Superior RBANS gains in all 5 domains (p <0.05) Symptom-cognition correlation: Y-BOCS↓ linked to RBANS↑ only in EEG group (*r = 0.43, p = 0.007*)	Non-personalized
Hawley et al. (2021)	RCT	EEG-NF (Muse)	71 OCD patients (36 Muse/36 waiting list control)	Frontal/Temporal (FP1, FP2, TP9, TP10) α (7.5–13 Hz), β (13–30 Hz)	Reduced OCD severity, improved mindfulness, altered a/p power (13–30 Hz) Symptom Reduction (Y-BOCS): Significant OCD symptom improvement in Muse group vs. waiting list group (medium effect size). Mindfulness (Five Factor Mindfulness Questionnaire (FFMQ) (Baer, Smith, Hopkins, Krietemeyer, & Toney, 2006)): Increased Non-Reactivity facet in Muse group. EEG Correlates: Increased α/β power (indicating reduced mind wandering) in Muse group. Temporal Latent Difference Score models showed increased α/β power and FFMQ Non-Reactivity predicted subsequent Y-BOCS reduction. No significant changes in δ/θ.	Non-personalized
Scheinost et al. (2014)	Uncontrolled trial	fMRI-NF	10 subclinical contamination anxiety/5 OCD patients	Orbitofrontal cortex/anterior prefrontal cortex (BA10)	Baseline functional connectivity (degree) in OFC/BA 10 predicted improvement in contamination anxiety symptoms after neurofeedback training. OCD subgroup (n = 5): Mean Y-BOCS decreased from 26.8 at baseline to 20.6 post-training (~24% mean improvement).	Personalized
Buyukturkoglu et al. (2015)	Case-series	fMRI-NF	3 OCD patients	Bilateral Anterior Insula	Behavioral Measures: Ecological Disgust Test: Reduced avoidance distance to real disgust objects (e.g., chewed gum, used toilet paper) during down-regulation in 2/3 patients. Picture Rating Test (VAS): Reduced negative valence and OCD symptom provocation ratings for disgust-inducing images during down-regulation in 2/3 patients (no change in arousal). Neural Measures: Successful down-regulation of anterior insula BOLD signal during neurofeedback training in all patients (varying degrees). Clinical Scales (pre/post): Y-BOCs: Mixed results (Patient 1: 12 → 11; Patient 2: 6 → 9; Patient 3: 36 → 33). STAI-State (Laux, 1981): Reduced anxiety in scanner for patient 1.	Personalized
Rance et al. (2023)	RCT	fMRI-NF	36 OCD patients(18 NF/18 sham)	Anterior Prefrontal Cortex (aPFC)	Active group had greater Y-BOCS reduction; delayed effects at follow-up Primary Clinical outcome: Significant group ×time interaction for Y-BOCS Total Score (*p = 0.04*), with greater reduction in active vs. sham group (max Δ = ~ 2 points). No significant change in symptom-specific Y-BOCS (focused on primary domain: contamination/washing or fear-of-harm/checking). Neural outcome: No improvement in aPFC self-regulation during control (transfer) tasks or neurofeedback runs.	Personalized

Studies are organized by modality, design, and protocol characteristics. Key findings include symptom outcomes referenced to validated scales (e.g., Yale-Brown Obsessive Scale [Y-BOCS], Padua Inventory). Protocol specificity indicates whether NF protocols were individualized to participant neurophysiology (personalized) or standardized across participants (non-personalized).

Abbreviations:NF = neurofeedback; RCT = randomized controlled trial; qEEG = quantitative electroencephalography; ICA = independent component analysis; SMR = sensorimotor rhythm; CBT = cognitive behavioral therapy; SRI = serotonin reuptake inhibitor; Y-BOCS = Yale-Brown Obsessive Compulsive Scale; RBANS = Repeatable Battery for the Assessment of Neuropsychological Status; FFMQ = Five Factor Mindfulness Questionnaire; VAS = visual analog scale; STAI = State-Trait Anxiety Inventory; aPFC = anterior prefrontal cortex.

## Appendix

**Table:** Abbreviation Table

Abbreviation	Full Term
ACC	Anterior Cingulate Cortex
BDD	Body Dysmorphic Disorder
BOLD	Blood Oxygen Level-Dependent
CBT	Cognitive-Behavioral Therapy
CSTC	Cortico-Striato-Thalamo-Cortical
DecNef	Decoded Neurofeedback
DLPFC	Dorsolateral Prefrontal Cortex
DVS	Dorsal Visual Stream
EEG	Electroencephalogram
EEG-NF	Electroencephalogram-based Neurofeedback
ERP	Exposure and Response Prevention
fMRI	Functional Magnetic Resonance Imaging
fMRI-NF	fMRI-based Neurofeedback
fNIRS	Functional Near-Infrared Spectroscopy
ICD-11	International Classification of Diseases, 11th Revision
NF	Neurofeedback
OCRDs	Obsessive-Compulsive and Related Disorders
OCD	Obsessive-Compulsive Disorder
OFC	Orbitofrontal Cortex
qEEG	Quantitative Electroencephalogram
ROI	Region of Interest
RDoC	Research Domain Criteria
SRI	Serotonin Reuptake Inhibitor
SMA	Supplementary Motor Area
SRIs	Serotonin Reuptake Inhibitors
tDCS	Transcranial Direct Current Stimulation
TMS	Transcranial Magnetic Stimulation
vmPFC	Ventromedial Prefrontal Cortex
Y-BOCS	Yale-Brown Obsessive-Compulsive Scale
